# Carfilzomib-associated renal toxicity is common and unpredictable: a comprehensive analysis of 114 multiple myeloma patients

**DOI:** 10.1038/s41408-020-00381-4

**Published:** 2020-11-03

**Authors:** Despina Fotiou, Maria Roussou, Charikleia Gakiopoulou, Erasmia Psimenou, Maria Gavriatopoulou, Magdalini Migkou, Nikolaos Kanellias, Ioanna Dialoupi, Evangelos Eleutherakis-Papaiakovou, Stavroula Giannouli, Christina Delavinia, Kostantinos Efstathiou, Sofoklis Kontogiannis, Evangelos Terpos, Meletios A. Dimopoulos, Efstathios Kastritis

**Affiliations:** 1 Department of Clinical Therapeutics, National and Kapodistrian University of Athens, School of Medicine, Athens, Greece; 2 Fist Department of Pathology, National and Kapodistrian University of Athens, Athens, Greece; 3 Second Department of Internal Medicine, Hematology Unit, National and Kapodistrian University of Athens, School of Medicine, Athens, Greece

**Keywords:** Myeloma, Adverse effects, Chemotherapy

## Abstract

Carfilzomib (CFZ) is a non-reversible proteasome inhibitor approved for the treatment of patients with relapsed and refractory myeloma (RRMM). Its use has been associated with cardiovascular toxicity but although recently a signal of clinically significant renal complications has also been identified, it is less extensively investigated. We analyzed data of 114 consecutive patients with RRMM who received CFZ-based regimens. Renal complications not related to MM progression were observed in 19 (17%) patients; thrombotic microangiopathy (TMA) was seen in 6 (5%) patients, albuminuria >1 gr/day in 7 patients (6%) and at least grade 3 acute kidney injury (AKI) which could not be otherwise explained in 6 patients (5%). A total of 15 patients discontinued CFZ and dosing was reinitiated at a lower level in one patient with AKI. Albuminuria was associated with focal segmental glomerulosclerosis in the renal biopsy (performed in a total of 6 patients). Renal complications during CFZ therapy are common, occur mostly early and are unpredictable. A potential effect of CFZ on the renal endothelium could be implicated in the pathogenesis of these complications and may also share common pathophysiology with cardiovascular effects of CFZ.

Carfilzomib (CFZ) is a next-generation selective and nonreversible proteasome inhibitor (PI), approved at different dosing schedules for patients with relapsed/refractory multiple myeloma (RRMM)^1,2^. Compared to bortezomib, carfilzomib has a different safety profile, with a low risk of neurotoxicity but higher for cardiovascular toxicity^2^. Renal clearance is not a significant pathway for carfilzomib elimination and no dose or treatment schedule adjustments are required in patients with renal impairment^3^ but renal toxicity has been described^4^. In addition, an association with thrombotic micorangiopathy (TMA) has been reported and has been included in the drug’s Summary of product characteristics. There is, however, limited characterization of the spectrum of renal toxicities associated with carfilzomib, and, given its potent antimyeloma activity and increasing use, a thorough characterization of these toxicities is clinically relevant. We report data for 114 consecutive, unselected, RRMM patients who were treated with carfilzomib-based regimens in our center (Department of Clinical Therapeutics, Athens), closely monitored and evaluated for renal complications, including renal biopsies. Renal events related to myeloma progression were not rated as carfilzomib-related. They were rated according to National Cancer Institute Common Terminology Criteria for Adverse Events, v4.03. Approval was obtained from the institutional review board/Ethics Committee of our hospital. Renal events of interest and discontinuation of carfilzomib as a result of progression of disease or for other reasons was treated as a competing event and the respective cumulative incidence function curves were plotted.

Patients’ characteristics are shown in Table 1. The median follow-up from the start of carfilzomib is 27 months, median duration of carfilzomib therapy was 5.5 months (IQR 3.2−11.5) and 28 (24.5%) patients continued carfilzomib therapy at the time of analysis. During treatment and up to 30 days after the last carfilzomib dose, 19 (17%) patients developed renal complications (not related to myeloma progression) (Fig. 1). These included TMA in 6 (5%) patients, albuminuria > 1 g/day with very low serum free light chains (sFLCs) or negative urine immunofixation in 7 (6%) patients and acute kidney injury (AKI) ≥ grade 3 in 6 (5%) patients which could not be otherwise explained. Median time from start of carfilzomib to development of any renal complication was 62 days (~2 months) (IQR 35−272) and in 15/19 patients carfilzomib was discontinued due to renal complications.

**Table 1:** Patient clinical characteristics.

	*N* = 114
Age, years, median (range)	70 (36–86)
Gender, male/female	60.5%/39.5%
Median baseline eGFR (ml/min/1.73 m^2^) (range)	78.42 (22.2–129)
Median baseline proteinuria (range) (g/24 h)	0.249 (0.034–8.1)
Comorbidities	
Hypertension	49 (43%)
Diabetes mellitus	23 (20%)
Peripheral angiopathy	21 (18%)
Coronary heart disease	7 (6%)
MM immunoglobulin type	
κLC	70 (614%)
λLC	39 (34.2%)
Non-secretory	5 (4.4%)
Previous HDM/ASCT	53 (47%)
Median number of previous treatments (range)	2 (1–7)
Bortezomib	78%
ImiD	73%
Anthracycline	27%
ASCT	46.5%
Carfilzomib dose (mg/m^2^)	
20/27	30%
20/36	11%
20/56	59%
Carfilzomib-dexamethasone (Kd)	75%
Kd+ Lenalidomide (KRd)	14%
Other CFz combinations	11%

*IMiD* immunomodulatory agent, *ASCT* autologous stem cell transplant.

**Fig. 1:**
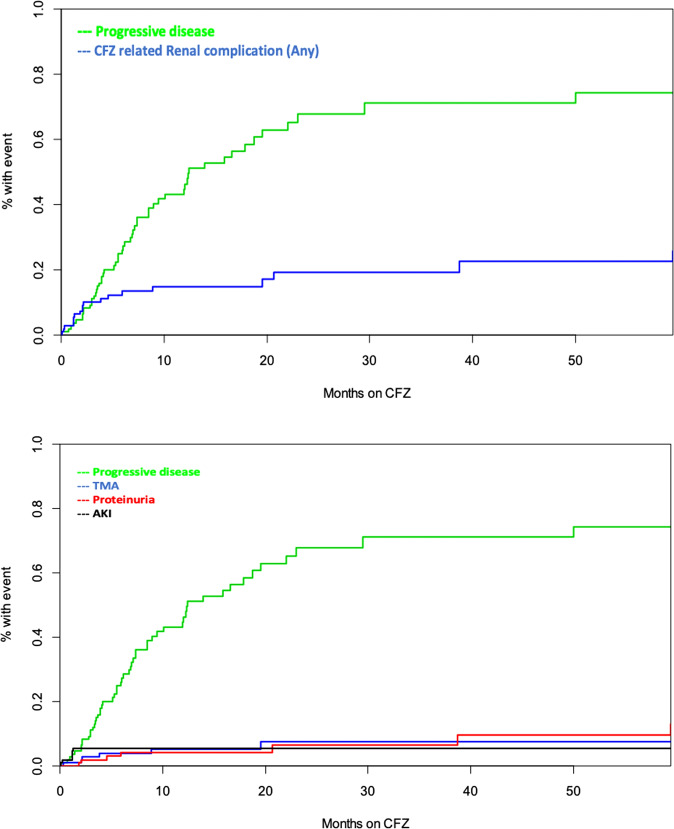
Renal complications and Carfilzomib treatment. Upper panel: Percentage of patients with progressive disease and any carfilzomib-related complications plotted over time of months on Carfilzomib (CFZ) treatment. Lower panel: Renal complications observed in MM patients. TMA thrombotic microangiopathy, AKI acute kidney injury.

Median time from carfilzomib start to TMA was 3 months (0.5–19.5). At diagnosis of TMA, median platelet counts were 20 × 10^9^/L (range 11–30), median hemoglobin 8 g/dL, median Lactate dehydrogenase (LDH) 449 IU/L (ULN < 225, range 371–619) and median blood schistocytes were 2.5% (range 2–6.5%). All six patients received plasmapheresis and steroids, rituximab was administered in one patient; no patient received eculizumab. Renal function and platelets recovered fully in five patients while one died of sepsis. No patient had progressive myeloma at the time of the event. ADAMTS-13 was evaluated in two patients and was within normal levels. No patient was re-exposed to carfilzomib after TMA.

Median time to development of proteinuria >1 g/d was 6 months (range 2–59 months). Median proteinuria was 3.7 g/d (range 1–4.5) and in all cases >90% of urine protein was albumin. All seven patients were in myeloma remission. Median eGFR was 53 mL/min/1.73 m^2^ (range 41–92 mL/min/1.73 m^2^) and one had proteinuria prior to carfilzomib, which was selective Bence−Jones proteinuria. Following carfilzomib interruption there was a gradual decrease of proteinuria to normal levels in two of the seven patients. Carfilzomib was resumed in one of the patients at a reduced dose with no recurrence of the proteinuria. Median time to CFZ-induced AKI was 1.1 months and median creatinine level was 2.5 mg/d. The injury was reversed in three patients who reinitiated treatment at lower doses.

Six patients with persistent renal dysfunction, with no obvious etiology and no contraindications had renal biopsies (Supplementary Table S1 and Supplementary Fig. S1); four had albuminuria and two had AKI. None had findings consistent with immunoglobulin-mediated pathology (cast nephropathy, monoclonal immunoglobulin deposition disease (MIDD) or amyloidosis) or pathology related to activation of the alternative complement pathway. The most consistent finding was a pattern of focal segmental glomerulosclerosis (FSGS) of various subtypes. Additionally, coexistent with the lesions of FSGS, in four renal biopsies, a pattern of acute and/or organized TMA was observed, with intraglomerular and/or arteriolar fibrin microthrombi and/or mucoid degeneration of the arteriolar/arterial wall and/or reduplication of the glomerular basement membranes with swelling of the endothelial cells.

We found no association of baseline proteinuria (immunoglobulin or albumin), sFLC, myeloma subtype, patient age, number of prior lines of therapy, the CFZ dose, carfilzomib regimen, baseline eGFR and prior history of cardiovascular disease, diabetes mellitus or hypertension with the occurrence of renal complications. At start of carfilzomib, 33 patients had an eGFR <60 mL/min/1.73 m^2^ and 18 (54.5%) improved their eGFR to >60 mL/min/1.73 m^2^.

Carfilzomib-associated renal complications were observed in 17% of patients in our cohort. They mostly occurred early, within the first 2–3 months in more than half of the patients, although albuminuria developed later (median time of 6 months). These complications seem unpredictable, as we were not able to identify any predictive factors. As a consequence, carfilzomib was discontinued in 15 (13%) patients, establishing carfilzomib-associated nephrotoxicity as a major cause of treatment delay and discontinuation. However, as previously demonstrated, renal impairment is often transient and carfilzomib was effective even for patients with renal dysfunction.

In contrast to previous reports, our evaluation of renal function was not limited to evaluation of creatinine levels alone^3,4^. Thus, we observed a relatively high rate of renal complications, by reporting renal adverse events that cover the whole spectrum of renal toxicities observed with carfilzomib. According to clinical trial data, grade 3 or higher events of AKI associated with CFZ are seen in approximately 3–5% of patients and in a recent meta-analysis of four phase II studies, the cumulative incidence of grade 3–5 renal toxicities was 8.3%^1,2,4^. In most datasets no distinction was made between renal impairment due to carfilzomib and secondary to disease progression but we were able to decipher these clinical conditions through detailed and meticulous evaluation in all cases.

The pathophysiology underlying carfilzomib-induced renal toxicity is not clear yet^3^. The mechanisms proposed range from prerenal insults, to tumor lysis-like phenomena, to biopsy-proven TM. The transient rise in creatinine, which is quite common, implies that the process might be prerenal or vascular and therefore reversible^5^. Endothelial dysfunction via an increased resting vasoconstricting tone and amplification of the spasmogenic effect of different agents could partly explain the renal effects of carfilzomib according to some animal and clinical data^6^. It has been demonstrated that proteasomal inhibition causes a shift in favor of endothelium-dependent vasodilation^7^ but inhibition of chymotrypsin-like (CT-L) subunit of the 20S proteasome by carfilzomib may be key to the vasoconstriction observed.

TMA was observed in several of our patients. The term TMA is used to describe a histopathological lesion characterized by microvascular occlusion secondary to endothelial injury and coagulation cascade activation. The clinical syndrome associated with this entity includes microangiopathic hemolytic anemia (MAHA), thrombocytopenia and end-organ damage^8^. In our cohort, clinically overt TMA was observed in six (5%) patients but was also a frequent pathological lesion in renal biopsies of patients that presented only with albuminuria, lacking the associated clinical features. Renal failure with biopsy-proven TMA in the absence of hematologic criteria has previously been reported. A recent case report describes renal-limited TMA in a patient who developed bevacizumab-induced proteinuria^9^. Drug-induced TMA (DITMA), as a clinical syndrome, is a relatively rare complication of several drugs, including PIs^10^. Two mechanisms have been postulated: immune-mediated antibody damage accounts for early events and direct toxicity which is time and dose-dependent and accounts for events at later timepoints^10,11^. Case reports have also highlighted the association between carfilzomib and AKI with evidence of underlying TMA (also biopsy-proven in some cases)^5,12^. In a case series on PI-induced TMA, patients had normal ADAMTS-13 levels and two underwent a biopsy confirming TMA^10^. Two mechanisms have been proposed for the direct nephrotoxic effects of carfilzomib. The first involves microvascular toxicity to the endothelium mediated by carfilzomib-induced reduction in VEGF levels (via NFκB inhibition)^13^. VEGF is essential for the functional and structural integrity of the glomerural endothelium and disruption of VEGF production can lead to TMA and podocytopathy, proteinuria and hypertension. The second mechanism involves a potential overactivation of the complement membrane attack complex. One group recently demonstrated increased deposition of C5b-9 on cultured endothelial cells when exposed to plasma of four patients during the acute phase of carfilzomib-induced TMA^14^.

Our data suggest that the spectrum of carfilzomib-related renal adverse effects extends beyond increase of serum creatinine, to TMA as a clinical syndrome, and albuminuria associated with FSGS but also with histopathological-only findings of TMA. Importantly, carfilzomib-induced renal toxicity is unpredictable. Thus, in our practice, we follow serum creatinine almost weekly and in case of significant creatinine increase we assess platelet counts and serum LDH. We also assess proteinuria and urine protein electrophoresis regularly, even in patients without Bence−Jones proteinuria at the start of carfilzomib therapy. Further investigation of the underlying mechanisms of carfilzomib-induced nephrotoxicity is needed, in order to predict and manage these complications and provide this active drug with safety.

## Supplementary information

Figure S1 + Table S1
