# Supplementary material for: Carfilzomib-associated renal toxicity is common and unpredictable: a comprehensive analysis of 114 multiple myeloma patients
Source: Blood Cancer J. 2020 Nov 3;10(11):109. doi: 10.1038/s41408-020-00381-4 (PMC7642386; doi:10.1038/s41408-020-00381-4)
Supplement: Supplementary file 1 — Figure S1 + Table S1 [file 41408_2020_381_MOESM1_ESM.docx]

Table S1: Table summarizing the features of Renal biopsies. G: Glomeruli, GG: Global Glomerulosclerosis, FSGS: Focal Segmental Glomerulosclerosis, nos : Not Otherwise Specified, TMA: Thrombotic Microangiopathy, IF: Interstitial Fibrosis, TA: Tubular Atrophy, Arteriolar Hyalinosis: 0= absent, 1=mild, 2= moderate, 3=severe (concentric), V: Vascular intimal thickening: 0: absent, 1: mild, 2:moderate, 3 severe, N: negative, NS: non specific

|  | **Age** | **Sex** | **No of G** | **GG** | **FSGS** | **FSGS**  **variant** | **TMA**  **lesions** | **IF** | **TA** | **AH** | **V** | **Immunofluorescence** |
| --- | --- | --- | --- | --- | --- | --- | --- | --- | --- | --- | --- | --- |
| 1 | 71 | M | 4 | 25% | Yes (25%) | perihilar | No | 35% | 35% | 1 | 0 | N or NS |
| 2 | 70 | M | 26 | 73% | Yes (7.6%) | perihilar | Yes | 40% | 35% | 3 | 3 | N or NS |
| 3 | 65 | M | 35 | 82.8% | Yes (5.7%) | cellular | Yes | >50% | >50% | 3 | 3 | N or NS |
| 4 | 70 | F | 24 | 58.3% | Yes (12.5%) | collapsing | Yes | 35% | 35% | 1 | 1 | N or NS |
| 5 | 67 | M | 12 | 16.6% | Yes (25%) | nos | Yes | 15% | 10% | 3 | 2 | N or NS |
| 6 | 60 | F | 24 | 25% | Yes (25%) | nos | No | 35% | 30% | 1 | 0 | N or NS |


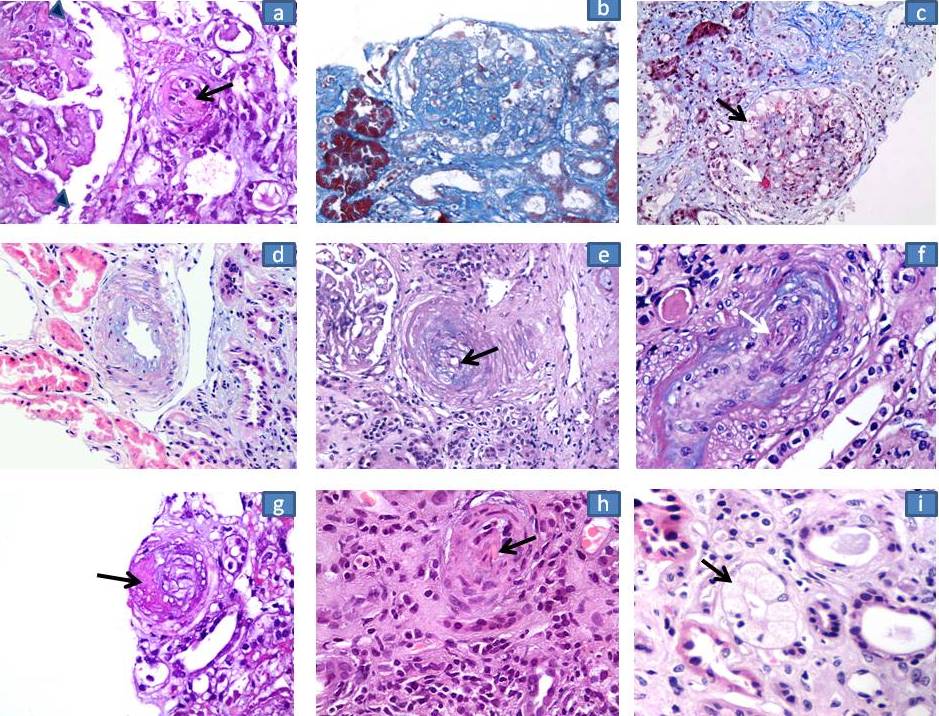


**Figure S1: Renal biopsy lesions, mainly of the TMA (Thrombotic Microangiopathy) pattern, observed in patients under carfilzomib. a.Arteriole with fibrin thrombus occluding the lumen (black arrow). It is also discerned part of a glomerulus exhibiting ischemic features and including intracapillary thrombi (arrowheads). (HE X400) b. Glomerulus with significant endothelial swelling occluding the capillary lumens. (Masson x200) c. Glomerulus with hyperplastic and swollen podocytes around collapsing sclerotic lesion. Note protein resorption droplets in the cytoplasm of podocytes (black arrow). There is also a fibrin thrombus (white arrow). (Masson x200) d. Interlobular artery with mild to moderate intimal mucoid degeneration. (HE X 200) e. Severe luminal narrowing (black arrow) due to myointimal hyperplasia, swelling and mucoid degeneration. The adjacent glomerulus shows significant ischemic changes with thickening and wrinkling of the glomerular basement membranes. (HE X200) f. Luminal occlusion (black arrows) due to circumferential myointimal thickening and mucoid degeneration of the wall of one arteriole with entrapped fragmented red blood cells and nuclear debris in the wall. (HE X400) g. Arteriole with fibrin thrombus (black arrow). (HE X 400) h. Arteriolar with concentric myointimal proliferation and fibrin insudation within the wall (black arrow). (HE X 400) i. Tubule showing isometric epithelial vacuolization. (HE X 400)**
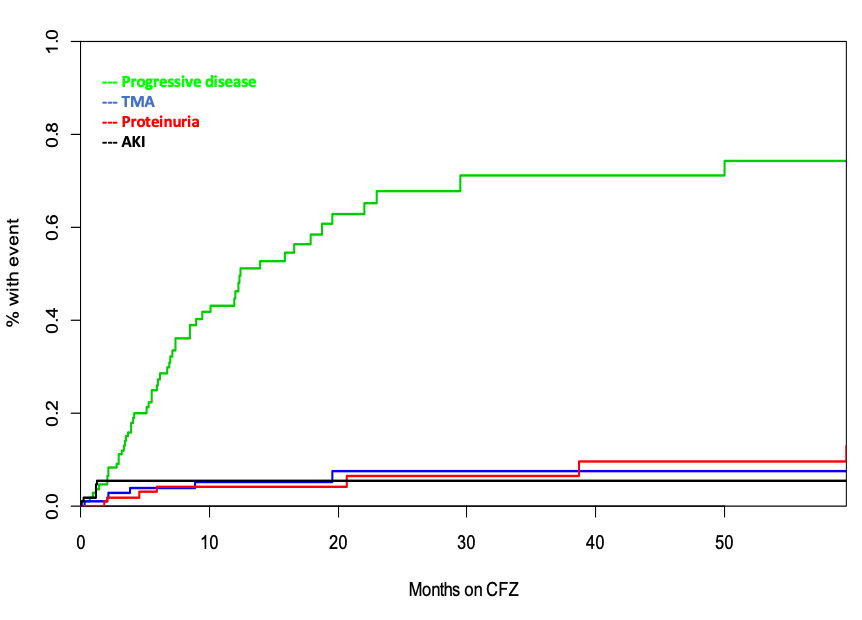

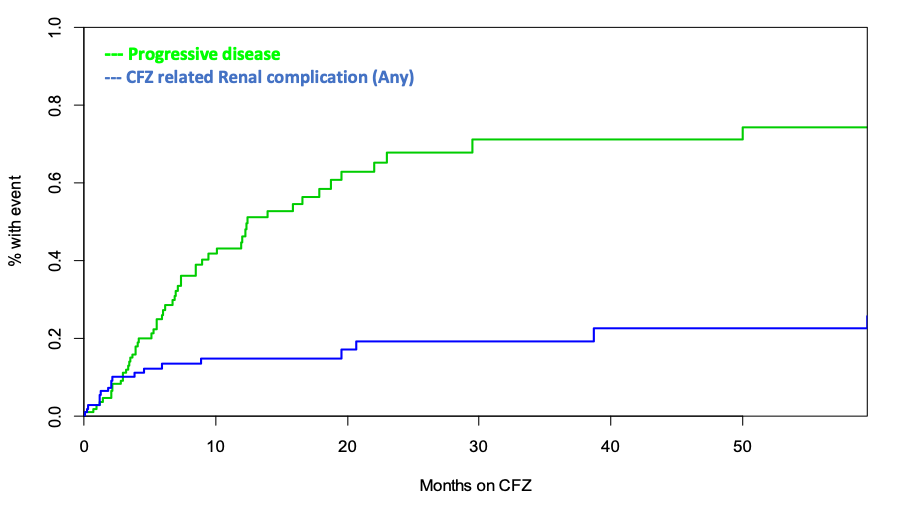


Figure : Percentage of patients with progressive disease and any Carfilzomib related complications plotted over time of months on CFZ treatment

Figure : Renal complications observed in MM patients. TMA: thrombotic microangiopathy; AKI: acute kidney injury
